# Comparative effectiveness and safety of ondansetron-based dual versus triple antiemetic regimens for chemotherapy-induced nausea and vomiting

**DOI:** 10.1097/MD.0000000000049639

**Published:** 2026-07-10

**Authors:** Xiang Yu, Ying Yuan, Guanglei Qiao, Wei Zheng, Zimei Liu, Jiuang Mao, Liping Yu, Zhoufeng Deng, Hongjian Lin, Jianjun Zhang

**Affiliations:** a Department of Oncology, The 903rd Hospital of Joint Logistic Support Force of the Chinese People’s Liberation Army, Hangzhou, Zhejiang Province, China; b Department of Oncology, Tongren Hospital, Shanghai Jiaotong University School of Medicine, Shanghai, China.

**Keywords:** aprepitant, chemotherapy-induced nausea and vomiting, CINV, dexamethasone, ondansetron, propensity score matching, retrospective cohort

## Abstract

Chemotherapy-induced nausea and vomiting remains a frequent complication of cytotoxic therapy and can compromise treatment tolerance and adherence. This retrospective propensity score-matched cohort study evaluated the comparative effectiveness and safety of ondansetron-based dual versus triple antiemetic regimens in patients receiving chemotherapy for malignant tumors. Consecutive patients treated between January 2022 and July 2023 who underwent at least 2 chemotherapy cycles were identified from hospital records. Patients received either a dual regimen (ondansetron plus dexamethasone or aprepitant) or a triple regimen (ondansetron plus dexamethasone plus aprepitant). Propensity score matching (1:1) was conducted using sex, age, tumor type, metastatic status, Karnofsky performance status score, and history of motion sickness to reduce confounding, yielding 319 matched pairs. Across the first 2 chemotherapy cycles, the triple regimen achieved higher control rates for nausea and vomiting than the dual regimen, with statistically significant differences in overall efficacy in both cycles. Subgroup analyses suggested the greatest benefit of triple therapy among patients receiving highly emetogenic chemotherapy, whereas both strategies achieved high efficacy in moderately emetogenic settings. Adverse reactions were more frequent with the triple regimen in the first cycle, while between-group differences were attenuated in the second cycle. These findings support a risk-adapted approach that prioritizes triple therapy for highly emetogenic regimens with attention to tolerability.

## 1. Introduction

Chemotherapy-induced nausea and vomiting (CINV) remains one of the most distressing adverse effects of cancer treatment and negatively affects quality of life and treatment adherence.^[1–5]^ To alleviate these side effects, ondansetron is currently a commonly used drug.^[6]^ It is a 5-hydroxytryptamine 3 (5-HT3) receptor antagonist that achieves antiemetic effects by blocking 5-HT3 receptors in the central nervous system and is widely used in the prevention and treatment of CINV.^[7]^ Although ondansetron can quickly take effect, it cannot solve the problems of gastrointestinal reactions and mucosal damage caused by chemotherapy drugs. In recent years, the exploration of combination drug strategies has become a research hotspot in CINV management. Clinical studies have confirmed that the combination of ondansetron and dexamethasone can significantly reduce the incidence of acute vomiting (complete remission rate of 50%–75%) in patients undergoing moderately or highly emetogenic chemotherapy (such as cisplatin and anthracyclines).^[8,9]^ In addition, the triple regimen of neurokinin-1 (NK1) receptor antagonists (such as aprepitant) combined with ondansetron and dexamethasone can increase the overall complete remission rate to 89%.^[10]^ However, the cost-effectiveness and suitable population of this regimen still need further verification. It is worth noting that different chemotherapy regimens (such as multiday chemotherapy or intrathecal administration) and cancer types (such as nonleukemic blood tumors) may affect the efficacy of the combined regimen.^[11,12]^ The existing evidence is mostly based on randomized controlled trials (RCTs), lacking systematic control of confounding factors. Current NCCN and MASCC/ESMO guidelines recommend complete response (CR), defined as no emesis and no rescue medication use, as the standard endpoint for evaluating antiemetic efficacy.

Although RCTs have established the efficacy of NK1 receptor antagonist-containing antiemetic regimens, evidence from routine clinical practice remains limited. Real-world studies provide complementary information regarding treatment effectiveness and safety in heterogeneous patient populations that are often underrepresented in clinical trials. Therefore, we conducted this propensity score-matched retrospective cohort study to evaluate the comparative effectiveness and safety of ondansetron-based dual and triple antiemetic regimens in daily oncology practice.

## 2. Subjects and methods

### 2.1. Subjects

This study was approved by the Ethics Committee of The 903rd Hospital of Joint Logistic Support Force of the Chinese People’s Liberation Army. A total of 917 cancer patients undergoing at least 2 cycles of chemotherapy between January 2022 and July 2023 were included. This study was approved by the Ethics Committee of our hospital. Inclusion criteria were: patients with malignancy confirmed histologically; patients who received cisplatin-based chemotherapy; patients with normal cognitive function; patients with an expected survival period >3 months; patients with Karnofsky performance status (KPS) scores >60; and patients who had not undergone surgery. Exclusion criteria were: patients with severe cardiovascular, cerebrovascular, liver, kidney, lung, immune hematopoietic system, and other serious diseases; patients with abnormal blood counts; patients with a history of hypersensitivity to aprepitant, dexamethasone, or ondansetron; patients with vomiting caused by reasons other than chemotherapy (CINV is often accompanied by a specific symptom pattern, such as recurrent episodes related to the chemotherapy cycle); and pregnant or lactating women.

### 2.2. Grouping and propensity score matching

Among the 917 patients, 446 received a dual regimen (ondansetron + 1 other antiemetic), and 471 received a triple regimen (ondansetron + 2 other antiemetics). Propensity score matching (PSM) was used to select patients based on 6 covariates, including gender, age, tumor type, metastasis, KPS score, and motion sickness history. Finally, there were 319 patients in each group.

### 2.3. Data collection

Demographic and clinical variables were extracted retrospectively from the electronic medical record system using a prespecified data abstraction form. The collected dataset comprised sex, age, and body weight at the time of chemotherapy initiation, as well as oncologic characteristics including primary tumor type and metastatic status. Functional status was assessed using the KPS score documented before each treatment cycle. In addition, a history of motion sickness was recorded based on admission notes and prior medical history entries. All variables were retrieved from baseline assessments preceding antiemetic administration, and data completeness and internal consistency were verified through cross-checking of medical, nursing, and chemotherapy order records.

### 2.4. Chemotherapy regimens

All patients received different chemotherapy regimens on days 1 to 3 of chemotherapy, for a total of 2 chemotherapy cycles. Chemotherapy regimens were chosen based on tumor type and staging: lung cancer: etoposide + cisplatin, paclitaxel + cisplatin, or pemetrexed + cisplatin; breast cancer: cyclophosphamide + epirubicin or cyclophosphamide + epirubicin + paclitaxel; colorectal cancer: capecitabine + oxaliplatin or oxaliplatin + leucovorin + 5-fluorouracil; hematologic malignancies: daunorubicin + cytarabine or cyclophosphamide + doxorubicin + vincristine + prednisone; gynecologic tumors: paclitaxel + carboplatin or paclitaxel + cisplatin.

### 2.5. Antiemetic regimens

The dual regimens consisted of ondansetron combined with any other antiemetic drug of a different mechanism (dexamethasone or aprepitant). The triple regimen involved the combination of ondansetron, dexamethasone, and aprepitant. Patients in the dual regimen group were administered ondansetron hydrochloride injection (Qilu Pharmaceutical Co., Ltd., SFDA approval no. H10970064, 8 mg, intravenously) or ondansetron hydrochloride tablets (Qilu Pharmaceutical Co., Ltd., SFDA approval no. H10970063, 8 mg, orally). Moreover, dexamethasone sodium phosphate injection (Chengdu Tiantaishan Pharmaceutical Co., Ltd., SFDA approval no. H51020513, 7.5 mg/3.75 mg intravenously, once a day), or aprepitant capsules (Qilu Pharmaceutical Co., Ltd., SFDA approval no. H20203321, 125 mg/80 mg, orally, once a day) was given. Patients in the triple regimen group received the same dosages and administration routes as the dual regimen group but were subjected to all 3 drugs simultaneously.

### 2.6. Assessment criteria

Antiemetic efficacy: CR: no emetic episodes and no use of rescue antiemetic medication during the assessment period, according to NCCN and MASCC/ESMO guideline recommendations. CR was considered the primary efficacy endpoint because it is the most widely accepted outcome measure in contemporary antiemetic clinical trials. Partial response: vomiting 1 to 2 times/day. Minor response: vomiting 3 to 5 times/day. No response: vomiting >5 times/day. Overall efficacy = (CR + partial response)/total cases × 100%. Nausea efficacy evaluation criteria: grade 0: no nausea. Grade I: presence of nausea but does not affect eating or daily life. Grade II: presence of nausea that impacts eating and daily life. Grade III: severe nausea leading to a bedridden status. Efficacy rate = (grade 0 + grade I)/total cases × 100%. Vomiting efficacy evaluation criteria: grade 0: no vomiting. Grade I: vomiting 1 to 2 times/day, no impact on eating or daily life. Grade II: vomiting 3 to 5 times/day, impacts eating and daily life. Grade III: more than 5 vomiting episodes/day, uncontrollable, severely impacts eating and daily life. Efficacy rate = (grade 0 + grade I)/total cases × 100%. Adverse reactions: incidence and types of side effects were recorded.

### 2.7. Statistical analysis

Statistical analysis was conducted using Statistical Package for the Social Sciences 26.0 software. Quantitative data conforming to a normal distribution were expressed as mean ± standard deviation and comparisons between the 2 groups were performed using the *t* test. Quantitative data not conforming to a normal distribution were expressed as median and quartiles (M[P25, P75]), and comparisons between the 2 groups were conducted using the nonparametric Wilcoxon test. Categorical data were presented as frequencies and percentages (%), and comparisons between the 2 groups were made using the χ^2^ test. A *P* < .05 was considered statistically significant. The “PSM” function in Statistical Package for the Social Sciences software was utilized, with baseline parameters showing statistically significant differences between the 2 groups as covariates for scoring and matching. The nearest neighbor matching method was chosen for 1:1 matching of covariates, with a caliper value of 0.1. Because this was a retrospective real-world study, no prospective sample size calculation was conducted. However, a post hoc power analysis was performed based on the primary endpoint (overall nausea control efficacy). With 319 matched patients in each group and the observed efficacy difference between groups, the statistical power exceeded 90% at a two-sided significance level of 0.05.

## 3. Results

### 3.1. Baseline characteristics before and after PSM

We retrospectively analyzed the clinical data of 917 cancer patients treated with ondansetron-based combination regimens. Among them, 446 patients (48.64%) received the dual regimens, and 471 (51.36%) received the triple regimens. Before PSM, there were statistically significant differences in gender, age, tumor type, metastasis status, KPS score, and history of motion sickness between the 2 groups (*P* *>* .05; Table 1). Through 1:1 PSM, 319 matched pairs were successfully created. After matching, no statistically significant differences in gender, age, tumor type, metastasis status, KPS score, and history of motion sickness were observed between the 2 groups (*P* *>* .05; Table 1).

**Table 1:** Baseline characteristics before and after PSM.

Factors	Before matching				After matching			
	Dual group (n = 446)	Triple group (n = 471)	Test statistic	*P* value	Dual group (n = 319)	Triple group (n = 319)	Test statistic	*P* value
Gender			χ^2^ = 4.483	.034			χ^2^ = 0.252	.874
Male (%)	231 (51.8)	210 (44.6)			149 (46.7)	146 (45.8)		
Female (%)	215 (48.2)	361 (55.4)			170 (53.3)	173 (54.2)		
Age (yr)	56.0 ± 10.5	54.0 ± 10.7	*t* = 2.823	.005	55.0 ± 10.9	53.7 ± 10.8	*t* = 1.1.542	.123
Weight (kg)	62.7 ± 9.3	62.1 ± 10.5	*t* = 0.496	.356	62.2 ± 9.2	61.8 ± 9.5	*t* = 0.583	.560
Tumor types (%)			χ^2^ = 12.969	.024			χ^2^ = 0.794	.977
Lung cancer	84 (18.8)	114 (24.2)			64 (20.1)	60 (18.8)		
Breast cancer	86 (19.3)	113 (24.0)			74 (23.2)	80 (25.1)		
Colorectal cancer	111 (24.9)	83 (17.6)			61 (19.1)	64 (20.1)		
Gynecologic tumors	37 (8.3)	29 (6.2)			18 (5.6)	20 (6.3)		
Hematologic tumors	25 (5.6)	22 (4.7)			20 (6.3)	19 (6.0)		
Other solid tumors	103 (23.1)	110 (23.4)			82 (25.7)	76 (23.8)		
Metastasis status			χ^2^ = 5.788	.016			χ^2^ = 0.109	.741
Metastatic	278 (62.3)	330 (70.1)			208 (65.2)	203 (63.6)		
Nonmetastatic	168 (37.7)	141 (29.9)			111 (34.8)	116 (36.4)		
KPS scores			–	<.001			–	.556
90–100	98 (22.0)	206 (43.7)			91 (28.5)	94 (29.5)		
80–89	176 (39.5)	187 (39.7)			143 (44.8)	148 (46.4)		
70–79	120 (26.9)	72 (15.3)			78 (24.5)	71 (22.3)		
60–69	47 (10.5)	4 (0.8)			5 (1.6)	4 (1.3)		
50–59	3 (0.7)	2 (0.4)			2 (0.6)	2 (0.6)		
Under 50	2 (0.4)	0			0	0		
History of motion sickness	63 (14.1)	42 (8.9)	χ^2^ = 11.749	<.001	42 (13.2)	39 (12.2)	χ^2^ = 0.177	.674

KPS = Karnofsky performance status, PSM = propensity score matching.

### 3.2. Comparison of the overall nausea control efficacy rates between the 2 groups

After the first chemotherapy cycle, the overall nausea control efficacy rates were 61.1% for the dual regimen group and 80.5% for the triple regimen group (χ^2^ = 28.238, *P* < .001; Table 2). After the second chemotherapy cycle, the overall nausea control efficacy rates were 71.5% for the dual regimen group and 84.0% for the triple regimen group (χ^2^ = 13.778, *P* < .001; Table 2). These results indicate that the nausea control efficacy rates for the ondansetron triple regimens show higher efficacy than the dual regimens.

**Table 2:** Comparison of nausea efficacy rates between the 2 groups.

Efficacy	Dual regimen group (n = 319)	Triple regimen group (n = 319)	χ^2^	*P* value
First cycle				<.001
Grade 0	104 (32.6)	173 (54.2)		
Grade I	91 (28.5)	84 (26.3)		
Grade II	115 (36.1)	40 (12.5)		
Grade III	9 (2.8)	22 (6.9)		
Efficacy rate	61.1%	80.5%	28.238	<.001
Second cycle				<.001
Grade 0	130 (40.8)	180 (56.4)		
Grade I	98 (30.7)	88 (27.6)		
Grade II	86 (27.0)	29 (9.1)		
Grade III	5 (1.6)	22 (6.9)		
Efficacy rate	71.5%	84.0%	13.778	<.001

### 3.3. Comparison of nausea control efficacy in different tumor types treated with ondansetron

In the first cycle, the nausea control efficacy in lung cancer, colorectal cancer, breast cancer, gynecologic tumors, and hematologic tumors after treating with ondansetron was 77.4%, 68.8%, 73.4%, 81.6%, and 59% (χ^2^ = 10.553, *P* = .061; Table 3). In the second cycle, the nausea control efficacy in lung cancer, colorectal cancer, breast cancer, gynecologic tumors, and hematologic tumors after treating with ondansetron was 83.9%, 79.2%, 79.9%, 92.1%, and 61.5% (χ^2^ = 18.818, *P* = .002; Table 3). These results indicate that the nausea control efficacy rates after treating with ondansetron are best for gynecologic tumors and poorest for hematologic tumors.

**Table 3:** Comparison of nausea control efficacy in different tumor types treated with ondansetron.

Efficacy	Lung cancer (n = 124)	Colorectal cancer (n = 125)	Breast cancer (n = 154)	Gynecologic tumors (n = 38)	Hematologic tumor (n = 39)	χ^2^	*P* value
First cycle							.767
Grade 0	48 (38.7)	60 (48.0)	62 (40.3)	18 (47.4)	14 (35.9)		
Grade I	48 (38.7)	26 (20.8)	51 (33.1)	13 (34.2)	9 (23.1)		
Grade II	22 (17.7)	30 (24.0)	40 (26.0)	6 (15.8)	16 (41.0)		
Grade III	6 (4.8)	9 (7.2)	1 (0.6)	1 (2.6)	0		
Efficacy rate	77.4%	68.8%	73.4%	81.6%	59.0%	10.553	.061
Second cycle							.187
Grade 0	58 (46.8)	68 (54.4)	70 (45.5)	21 (55.3)	14 (35.9)		
Grade I	46 (37.1)	31 (24.8)	53 (34.4)	14 (36.8)	10 (25.6)		
Grade II	15 (12.1)	18 (14.4)	31 (20.1)	2 (5.3)	15 (38.5)		
Grade III	5 (4.0)	8 (6.4)	0	1 (2.6)	0		
Efficacy rate	83.9%	79.2%	79.9%	92.1%	61.5%	18.818	.002

### 3.4. Comparison of nausea prevention efficacy by emetogenic risk level

Patients undergoing highly emetogenic chemotherapy achieved significantly better nausea prevention efficacy with the triple regimen compared with the dual regimen (Table 4). For patients with low and moderate emetogenic chemotherapy, both regimens achieved efficacy rates above 80%, except for the 75.2% efficacy of the dual regimen in cycle 1 (Table 4).

**Table 4:** Comparison of nausea prevention efficacy by emetogenic risk level.

Regimens	Efficacy	Low risk	Moderate risk	High risk	*P*
Dual		N = 7	N = 109	N = 203	
	Cycle 1 efficacy rate (%)	85.7%	75.2%	52.7%	<.001
	Cycle 2 efficacy rate (%)	85.7%	88.5%	62.5%	<.001
Triple		N = 2	N = 118	N = 199	
	Cycle 1 efficacy rate (%)	100%	89.0%	75.4%	.006
	Cycle 2 efficacy rate (%)	100%	89.8%	80.0%	.042

### 3.5. Comparison of the overall vomiting control efficacy rates between the 2 groups

After the first chemotherapy cycle, the overall vomiting control efficacy rates were 70.6% for the dual regimen group and 80.6% for the triple regimen group (χ^2^ = 8.154, *P* = .004; Table 5). After the second chemotherapy cycle, the overall nausea control efficacy rates were 75.5% for the dual regimen group and 84.0% for the triple regimen group (χ^2^ = 6.568, *P* = .010; Table 5). These results indicate that the vomiting control efficacy rates for the ondansetron triple regimens show higher efficacy than the dual regimens.

**Table 5:** Comparison of vomiting control efficacy between the 2 groups.

Efficacy	Dual regimen group (n = 319)	Triple regimen group (n = 319)	χ^2^	*P* value
First cycle				<.001
Grade 0	145 (45.5)	199 (62.4)		
Grade I	80 (25.1)	58 (18.2)		
Grade II	87 (27.3)	42 (13.2)		
Grade III	7 (2.2)	20 (6.3)		
Efficacy rate	70.6%	80.6%	8.154	.004
Second cycle				<.001
Grade 0	159 (49.8)	199 (62.4)		
Grade I	82 (25.7)	69 (21.6)		
Grade II	71 (22.3)	30 (9.4)		
Grade III	7 (2.2)	21 (6.6)		
Efficacy rate	75.5%	84.0%	6.568	.010

### 3.6. Comparison of the adverse reactions between the 2 groups

The overall incidence of adverse reactions in the triple regimen group was higher than in the dual regimen group during both the first and second cycles (*P* < .05; Table 6). In the first cycle, the adverse reactions in the triple regimen group were significantly higher than those in the dual regimen group (6.6% vs 2.8%, *P* < .05; Table 6). However, in the second cycle, the difference in adverse reaction rates between the 2 groups was not statistically significant (*P* > .05; Table 6).

**Table 6:** Comparison of the adverse reactions between the 2 groups.

Safety	Dual regimen group (n = 319)	Triple regimen group (n = 319)	χ^2^	*P* value
First cycle				
Total adverse reactions	9 (2.8)	21 (6.6)	4.232	.040
Constipation	6 (1.9)	16 (5.0)	3.813	.051
Fatigue	3 (0.9)	6 (1.9)	–	.505
Dizziness	2 (0.6)	4 (1.3)	–	.686
Dry mouth	1 (0.3)	7 (2.2)	–	.069
Second cycle				
Total adverse reactions	11 (3.4)	14 (4.4)	0.167	.683
Constipation	8 (2.5)	9 (2.8)	0	1
Fatigue	1 (0.3)	5 (1.6)	–	.217
Dizziness	2 (0.6)	3 (0.9)	–	1
Dry mouth	3 (0.9)	6 (1.9)	–	.505

## 4. Discussion

Chemotherapy is one of the most commonly used treatments for cancer. However, chemotherapy often leads to adverse reactions such as nausea and vomiting, which severely affect the quality of life of patients.^[13]^ The correct use of antiemetic drugs can help improve the effectiveness of chemotherapy and enhance patients’ adherence to treatment regimens.^[14]^ Because of the complex mechanism of gastrointestinal adverse reactions caused by chemotherapy, monotherapy has limitations, and combining multiple treatment methods can often achieve more significant results.^[15]^ Current National Comprehensive Cancer Network (NCCN) and Multinational Association of Supportive Care in Cancer/European Society for Medical Oncology (MASCC/ESMO) antiemetic guidelines recommend a 3-drug regimen consisting of a 5-HT3 receptor antagonist, dexamethasone, and an NK1 receptor antagonist for patients receiving highly emetogenic chemotherapy. Our findings are consistent with these recommendations and support the clinical value of triple antiemetic prophylaxis in routine practice. Our results are also broadly consistent with previous RCTs, including the TRIPLE study and phase III aprepitant-based trials, which demonstrated superior CR rates with NK1 antagonist-containing regimens compared with dual antiemetic strategies.

Ondansetron, a first-generation 5-HT3 receptor antagonist, exhibits potent antiemetic effects and is recommended for the prevention and treatment of nausea and vomiting associated with chemotherapy, radiotherapy, and postoperative recovery. However, its short half-life necessitates frequent dosing.^[16]^ Dexamethasone, a commonly used corticosteroid, inhibits the production and release of peripheral and central 5-HT, demonstrating a long-lasting, effective, selective, and safe profile.^[17,18]^ With a half-life of 36 to 54 hours, dexamethasone can compensate for ondansetron’s short duration of action when used concomitantly.^[19]^ Aprepitant, a NK1 receptor antagonist, effectively blocks the action sites of substance P or NK receptors, thereby suppressing chemotherapy-induced gastrointestinal reactions.^[20]^ It provides lasting antiemetic effects with a half-life of 9 to 13 hours and is effective in preventing gastrointestinal reactions caused by highly emetogenic chemotherapy regimens.^[21]^

This study demonstrates that among patients subjected to highly emetogenic chemotherapy, those who received the triple-drug regimen exhibited a higher prophylactic effect against nausea and vomiting compared with those who received the double-drug regimen. These findings suggest an association between triple antiemetic therapy and improved CINV control among patients receiving highly emetogenic chemotherapy. These findings are consistent with the results reported by Zhang et al.^[22]^ However, it should be noted that the triple-drug regimen has a higher overall incidence of adverse reactions, requiring careful monitoring and management in clinical practice. The superiority of the triple regimen was particularly evident during the delayed phase of CINV, which is biologically plausible because substance P-mediated NK1 receptor signaling plays a predominant role during this period. The addition of aprepitant may therefore provide enhanced protection against delayed CINV.

For patients subjected to moderately emetogenic chemotherapy, both the dual and triple regimens demonstrated efficacy rates exceeding 80%, indicating their effectiveness for this patient group. It is particularly noteworthy that in the first cycle, the dual regimen achieved a 75.2% efficacy rate for nausea control, which, although slightly below 80%, remains within an acceptable range. Therefore, treatment options can be tailored to the individual patient’s circumstances and preferences, offering a more personalized therapeutic approach.

This study also underscores the varying responses of different cancer types to the combination therapy with ondansetron. Patients with gynecological tumors demonstrated the highest therapeutic effect, and existing research has confirmed the clinical value of ondansetron in preventing nausea and vomiting caused by chemotherapy for gynecological cancers.^[23]^ Conversely, hematological malignancies showed the lowest efficacy, which may be related to the biological characteristics of different cancer types and the mechanisms of the drugs used. These findings highlight the importance of considering individual patient differences based on cancer type in clinical practice.

Furthermore, we observed that after the first treatment cycle, the total incidence of adverse reactions in the triple therapy group was significantly higher. This could be attributed to the complexity and interactions of the drugs in the triple therapy regimen. However, after the second cycle, there was no statistically significant difference in the overall adverse reaction rate between the 2 groups, indicating that patients’ tolerance to the drugs may improve during treatment. Constipation was the most common adverse reaction, deserving clinical attention and management.

This study also has several limitations. First, the retrospective observational nature of this study introduces an inherent risk of selection bias and information bias. Although PSM was performed to balance measured baseline variables, treatment allocation was not randomized and therefore may still be influenced by physician preference, patient characteristics, and clinical practice patterns. Another limitation is that the retrospective database did not contain sufficiently detailed time-specific records to allow separate analyses of acute (0–24 hours) and delayed (24–120 hours) CINV. Since these 2 phases are mediated by partially distinct biological mechanisms and may respond differently to antiemetic therapy, future prospective studies should incorporate guideline-recommended phase-specific assessments. Furthermore, residual confounding cannot be completely eliminated because several potentially relevant variables were unavailable in the electronic medical records, including alcohol consumption history, anxiety status, previous CINV experience, anticipatory nausea and vomiting, concomitant medications, and socioeconomic factors. Therefore, causal inferences should be interpreted with caution.

## 5. Conclusion

In conclusion, the triple regimen combined with ondansetron was associated with improved efficacy in preventing nausea and vomiting induced by highly emetogenic chemotherapy, although with a higher incidence of adverse reactions. For patients undergoing moderately emetogenic chemotherapy, both dual and triple regimens show good efficacy in preventing nausea and vomiting. It is crucial to develop personalized treatment plans based on individual patients’ conditions and risks of adverse reactions. In clinical practice, ondansetron-based combination regimens may provide clinical benefit for improving chemotherapy-related nausea and vomiting. However, it is imperative to closely monitor and manage adverse reactions, and tailor treatment approaches according to cancer types for more precise therapy.

## Author contributions

**Conceptualization:** Xiang Yu, Ying Yuan, Guanglei Qiao, Wei Zheng, Zimei Liu, Jiuang Mao, Liping Yu, Zhoufeng Deng, Hongjian Lin, Jianjun Zhang.

**Data curation:** Xiang Yu, Ying Yuan, Guanglei Qiao, Wei Zheng, Zimei Liu, Jiuang Mao, Liping Yu, Zhoufeng Deng, Hongjian Lin, Jianjun Zhang.

**Formal analysis:** Xiang Yu, Ying Yuan, Guanglei Qiao, Wei Zheng, Zimei Liu, Jiuang Mao, Liping Yu, Zhoufeng Deng, Hongjian Lin, Jianjun Zhang.

**Funding acquisition:** Jianjun Zhang.

**Investigation:** Jianjun Zhang.

**Writing – original draft:** Jianjun Zhang.

**Writing – review & editing:** Jianjun Zhang.
